# A Business-to-Business Collaboration System That Promotes Data Utilization While Encrypting Information on the Blockchain

**DOI:** 10.3390/s22134909

**Published:** 2022-06-29

**Authors:** Hiroaki Nasu, Yuta Kodera, Yasuyuki Nogami

**Affiliations:** 1 Graduate School of Natural Science and Technology, Okayama University, Okayama 700-8530, Japan; 2 Faculty of Natural Science and Technology, Okayama University, Okayama 700-8530, Japan; yuta_kodera@okayama-u.ac.jp (Y.K.); yasuyuki.nogami@okayama-u.ac.jp (Y.N.)

**Keywords:** business-to-business data collaboration, industrial supply chain, blockchain, homomorphic encryption

## Abstract

Ensuring the reliability of data gathering from every connected device is an essential issue for promoting the advancement of the next paradigm shift, i.e., Industry 4.0. Blockchain technology is becoming recognized as an advanced tool. However, data collaboration using blockchain has not progressed sufficiently among companies in the industrial supply chain (SC) that handle sensitive data, such as those related to product quality, etc. There are two reasons why data utilization is not sufficiently advanced in the industrial SC. The first is that manufacturing information is top secret. Blockchain mechanisms, such as Bitcoin, which uses PKI, require plaintext to be shared between companies to verify the identity of the company that sent the data. Another is that the merits of data collaboration between companies have not been materialized. To solve these problems, this paper proposes a business-to-business collaboration system using homomorphic encryption and blockchain techniques. Using the proposed system, each company can exchange encrypted confidential information and utilize the data for its own business. In a trial, an equipment manufacturer was able to identify the quality change caused by a decrease in equipment performance as a cryptographic value from blockchain and to identify the change one month earlier without knowing the quality value.

## 1. Introduction

In order to promote Society 5.0, the Industrial Internet of Things, Industry 4.0, and so forth, it is important to connect and share data so that all IoT devices and all members can trust them. Blockchain (BC) technology is attracting attention as an advanced tool. Blockchain is a digital ledger for record-keeping over peer-to-peer (P2P) networks [1,2]. It is decentralized and dispersed in nature with tamper-resistant and tamper-evident features [3,4,5]. Each peer stores a copy of the blockchain and verifies the validity of the stored data, such that no peers can tamper with the data. For example, in the blockchain, all money in and out is stored in the block as historical data and connected in chronological order. The hash value of the previous block is included in the next block. Even if a peer participating in the network falsifies the data of a certain block, the hash value of the block will change, so it will not match the hash of the next block connected to it. Therefore, the fraud of the peer can be immediately revealed.

The concept of blockchain technology was derived from Bitcoin cryptocurrency, and then it spread to the financial field. This is still the main field in which blockchain technology is used [6,7]. Currently, in addition to the financial field, the blockchain technique is used in smart homes [8], smart cities [9,10], smart agriculture [11,12], smart power grids [13], smart transportation and automotives [14], smart healthcare [15,16], IoT networks [17,18], and security privacy [19]. In the smart-manufacturing field, much research has been conducted related to the certificate of origin, procurement, production, inspection, logistics, and sales [20,21,22,23,24,25,26,27]. In the business-to-business (B2B) manufacturing industry, the demands for high-quality product development and plan optimization can be met by sharing manufacturing data among companies in the supply chain (SC) [28,29,30,31]. To meet these demands, manufacturers need an environment where the data shared by the company cannot be tampered with. Additionally, each company’s manufacturing and inspection process data must be connected throughout the SC, from materials to products. BC technology is one an effective approach for building such an environment. However, production or inspection data collaboration using BC has not progressed sufficiently among companies in the industrial SC. One of the main reasons is that production and inspection data are highly confidential and therefore difficult to disclose to other companies (e.g., product quality data, equipment data, and order-shipping data) [32].

There are movements to improve data infrastructure beyond companies, mainly in Europe [33,34]. Attention is also focused on efforts to optimize the entire SC by constructing twin digital platforms. However, no matter how much infrastructure and how many platforms are built, in terms of B2B manufacturing data collaboration, it will be difficult to freely link highly confidential data unless two companies can agree on a contract. With data collaboration based on a contract between two companies, it is difficult to build an ecosystem in which data is widely and freely linked among companies. Even if a consortium were to be formed, as long as there is competition among companies, companies would not be able to safely disclose their data, which is the source of their revenue.

Regarding this issue, refs. [35,36] proposed methods for privacy protection for the IoT by combining encryption with BC or IOTA. Ref. [37] proposed an algorithm of the privacy-preserving OLPA for big data analysis. However, there is another major reason why data collaboration between companies is not progressing; the merits of data collaboration in a concrete manufacturing scene are not readily apparent, and its implementation with respect to both business and technology is difficult. It is important to design the benefits of being able to put data into a BC while ensuring a company’s competitive advantage. The authors of [35,36,37] have not been able to propose a protocol in consideration of the utilization in business after encryption. In order to achieve B2B collaboration between companies in the industrial SC, it is important to have a protocol and a system that can both “protect data” and “utilize data in the manufacturing process”.

This paper proposes a B2B collaboration system using homomorphic encryption (HE) and BC techniques. Using the proposed system, each company in the industrial SC can exchange confidential information, such as quality data, in an encrypted manner and utilize the data in their own manufacturing. In addition, this paper shows a scenario, system architecture, and protocol for upstream companies to grasp changes in the manufacturing quality of downstream companies. In this scenario, this paper evaluates the merit of concrete data collaboration in actual business using the proposed system.

The paper is structured as follows: Section 2 describes the blockchain issue in data linkage, a scenario for realizing B2B collaboration in the production process in the SC, and the issue of numerical comparison while maintaining the encryption required in the scenario. Section 3 proposes a secure comparison protocol and a B2B collaboration system to resolve these issues. Section 4 demonstrates the usefulness of the proposed system and protocol in an actual business scenario, and Section 5 evaluates the safety of the proposed system.

## 2. Preliminaries

### 2.1. The Problem of BC Utilization and the Secure Approach in B2B Collaboration in the SC

**Figure 1:**
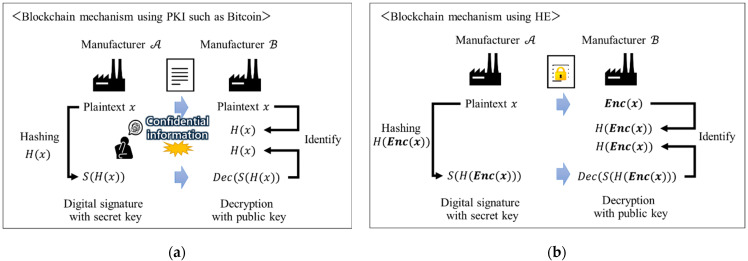
The issue of BC utilization and the secure approach in B2B collaboration. (**a**) Issue of BC utilization; (**b**) Secure approach in B2B collaboration.

In the B2B manufacturing industry, it is difficult for each company to disclose confidential information regarding its manufacturing know-how, even if it is a company that transacts in the SC. Blockchain mechanisms such as Bitcoin, which uses public key cryptography (PKI), require that plaintext be sent to identify a company (Figure 1a). Therefore, blockchains are not widely used for collaboration between companies in the manufacturing SC [32].

Regarding this issue, this paper proposes a secure B2B collaboration system in the SC that enables open data transfer and business coordination by combining blockchain technology and homomorphic encryption. In 1978, Ronald Rivest et al. suggested a proposal on the concept of homomorphic encryption [38]. Later, Craig Gentry proposed fully homomorphic encryption that computes an arbitrary number of additions and multiplications to encrypted data [39]. This scheme enables the programs for any desirable functionality, such as homomorphic property, to run on encrypted data and produce an encryption of the result. A partial homomorphic encryption exhibits either an additive or multiplicative homomorphism, but not both. In addition, the efficiency of some partial homomorphic encryption schemes is high enough for practical applications [40].

In a secure collaboration system that uses partial HE and blockchain techniques, each company in the SC can exchange confidential information using encrypted data and utilize it for their own business needs (Figure 1b). In this paper, as shown in Figure 1b, the encryption of plaintext x using HE is denoted as $Enc\left(x\right)$.

### 2.2. A B2B Collaboration Scenario on the Industrial SC

Let us consider a scenario in which A manufactures a product $P_{A}$ of quality $Q_{A}$ with equipment $E_{A}$ and delivers $P_{A}$ to B. B uses $P_{A}$ as a material to manufacture a product $P_{B}$ of quality $Q_{B}$ with equipment $E_{B}$. At this time, A wants to optimize the production plan by identifying the level of quality $Q_{B}$ that can be produced when the product $P_{A}$ is put into the equipment of B. Although B wants a stable supply of high-quality materials from A, it does not want to disclose its own manufacturing information because it is confidential. For this scenario, in this research, B sends $Enc\left(P_{B},\;Q_{B},\;E_{B}\right)$ to A using HE. Therefore, A can calculate its relationship and compatibility with $\left(P_{A},\;Q_{A},\;E_{A}\right)$ without knowing the specific product name $P_{B}$, quality $Q_{B}$, and equipment $E_{B}$, and it can formulate the optimum production plan for A.

In the field of chemistry, products are manufactured by reacting materials. Therefore, the impact on product quality caused by the physical properties of materials and the compatibility of equipment is important. The utilization of the proposed scenario in real businesses can be expected.

In regard to the above scenario, this paper focuses on quality data. A wants to catch the change of $Enc\left(Q_{B}\right)$ at time t and t+1, and if there is a big change, A will identify its own manufacturing factors, leading to optimum production. Therefore, in order to realize the proposed scenario, it is important to have a comparison protocol for the values of t and t+1 so as to encrypt them. In the protocol, a function to put quality data into the blockchain and a function to get them from the blockchain are also important for implementing the proposed scenario.

**Figure 2:**
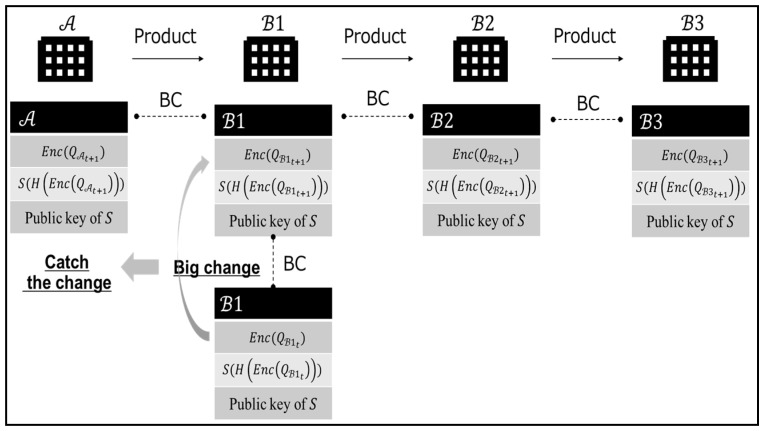
The final system of the B2B collaboration.

The final system for the B2B collaboration is shown in Figure 2. In this final system, manufacturing companies can chain data without disclosing their quality data, while also guaranteeing their identities using blockchain. Even if the encrypted quality data were to be tampered with by an attacker, the hash value of the encrypted quality data would not match the value after decrypting the signature. Therefore, tampering could be detected immediately. In addition, traceability in the SC is possible by including the lot number of each company’s products in the encryption. Under such a secure data linkage, A will be able to identify changes in the quality data of downstream companies in a timely manner and to utilize that information in its own manufacturing.

### 2.3. Conventional Comparison Protocol Using HE and Issues with B2B Collaboration

In 2016, Wu et al. proposed a comparison protocol based on Paillier cryptography, a kind of additive homomorphic encryption [41]. In the protocol, the client and server have the values x and y, respectively. Neither party learns anything else about the other party’s input.

In the protocol, suppose the binary representations of x and y are $x_{0},\;x_{1},\;\ldots ,\;x_{k-1}$ (*k* bits) and $y_{0},\;y_{1},\;\ldots ,\;y_{k-1}$ (*k* bits), respectively. Using the following proposition, x>y or x<y is determined.

**Proposition** **1.** 
x<y *if and only if there exists some index $i\in \left[k-1\right]$* *that satisfies Formula (1). x>y* *if and only if there exists some index $i\in \left[k-1\right]$* *that satisfies Formula (2).*

(1) $$x_{i}-y_{i}+1+3\sum_{j<i}\left(x_{j}⊕y_{j}\right)=0,$$

(2) $$x_{i}-y_{i}-1+3\sum_{j<i}\left(x_{j}⊕y_{j}\right)=0.$$

Here are the details. The client and server encrypt $x_{i}$ and $y_{i}$ with the public key, respectively. The client sends $Enc\left(x_{i}\right)$ to the server. The server calculates Formula (1) or (2) by substituting $Enc\left(x_{i}\right)$ and $Enc\left(y_{i}\right)\;$ and using plaintext $y_{i}$ for XOR. The client receives the calculation result, decrypts it with the secret key, and checks for zero. Therefore, the client can determine that x>y or x<y without disclosing the value of x to the server.

In the proposed scenario for B2B collaboration, B has both x and y of the quality data, and A has $Enc\left(x\right)$ and $Enc\left(y\right)$. Therefore, A cannot calculate XOR using the above formula, and this conventional protocol is difficult to apply to B2B collaboration in the SC. In addition, in order to realize the proposed scenario in an actual business, it is necessary to consider a system architecture, including business viewpoints and a comparison protocol, according to the architecture.

## 3. Proposal B2B Collaboration System on the SC

In [42], our previous work showed a secure comparison protocol for B2B collaboration in the SC at ICCE-TW2021. This paper shows the concrete system architecture required to implement the proposed scenario for the actual business. This paper also shows the usefulness of the proposal system by adding the evaluation results in specific business situations.

### 3.1. System Architecture of the B2B Collaboration

**Figure 3:**
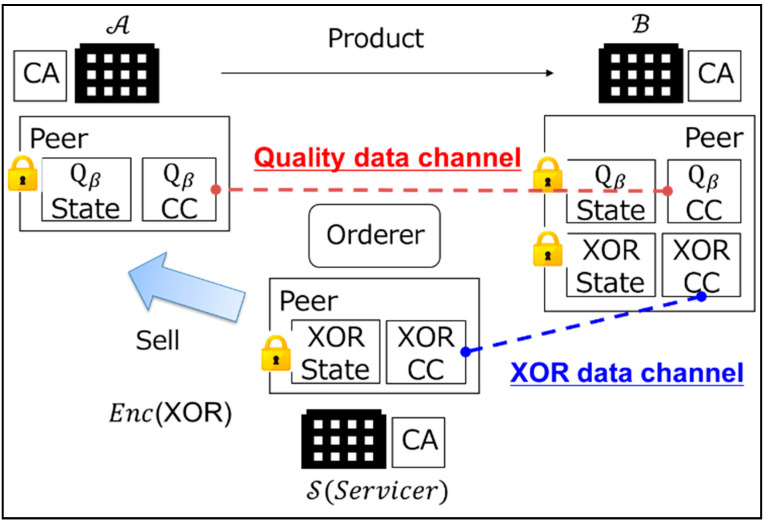
Proposed B2B collaboration system.

This paper shows the system architecture for implementing the proposed scenario in Figure 3. In a real business, it is necessary to have a servicer that provides value by exchanging data and guaranteeing the service level. In other words, the servicer is the company responsible for realizing the proposed scenario and the solution engineer who builds the data platform business. Therefore, the proposed system has a servicer S, as well as the manufacturers A and B. Each organization has at least one peer and certificate authority (CA) that manages the members of the organization, and the data is put into the blockchain by the orderer. Each peer has a state database that records the state of the data and a chaincode that holds the history of data transfers as a distributed ledger of the blockchain.

As shown in Figure 3, this paper proposes a multi-channel system architecture that separates the chaincode for the quality data and the XOR data. B has both x and y of the quality data and B puts $Enc\left(x\right)$ and $Enc\left(y\right)$ into the quality chaincode of B ($Q_{\beta }$ CC). A can get $Enc\left(x\right)$ and $Enc\left(y\right)$ from $Q_{\beta }$ CC. In the proposed scenario, the XOR data of B are essential for the calculation of Formulas (1) and (2). These are important key data, from both a technical and a business perspective. Therefore, in the proposed system architecture, B calculates x⊕y and puts $Enc\left(x⊕y\right)$ into the XOR chaincode (XOR CC). S can get $Enc\left(x⊕y\right)$ from XOR CC and sell $Enc\left(x⊕y\right)$ to A as a servicer. The reason that the servicer does not have the quality data is that the servicer and B may be in a competitive relationship. The servicer is only positioned to provide the key XOR data. Only A, which has a transaction with B in the SC, can grasp the change in quality.

There is another secondary reason for the architecture to have multiple channels with S. It is robust access control. If the product of B is manufactured from the materials of A and $A^{\prime }$, $A^{\prime }$ might get the big quality change in the material of A from $Q_{\beta }$ CC as a business opportunity and make an offer to B to replace an order for the material of A. Even if $A^{\prime }$ obtains the private key of A by some means, if S manages the XOR data and does not sell the data to $A^{\prime }$, $A^{\prime }$ cannot calculate Formula (1) or (2) without the XOR data.

### 3.2. Implementation of the Comparison Protocol to Realize the B2B Collaboration System

This paper proposes an improved secure comparison protocol for implementation in the B2B collaboration system using a variant of Wu’s protocol, which is based on Paillier cryptography. X and Y are the quality data (plaintexts) of B at time t and t+1, respectively.

In this proposed protocol, $Enc\left(x_{i}\right)$, $Enc\left(y_{i}\right)$, and $Enc(x_{i}⊕y_{i})$ are encrypted with Paillier cryptography [43]. In Paillier cryptography, message $m\in {\mathbb{Z}}_{n}$ is encrypted by (3) with integers n=p·q, $g=1+n\;\mathrm{mod}\;n^{2}$, where p and q are prime numbers of about 3000 bits, r is a random number 0<r<n $\in {\mathbb{Z}}_{n^{2}}^{*}$, and $\gcd\left(r,\;n\right)=1$. The public key is (n, g). The secret key is (p, q).

(3) $$C=g^{m}\cdot r^{n}\;\mathrm{mod}\;n^{2}$$

Decryption is carried out via the following formula using Carmichael’s theorem: $r^{n\lambda }\;\mathrm{mod}\;n^{2}=1$. Here, $\lambda =lcm\left(p-1,\;q-1\right)$ and a function $L\left(u\right)=\left(u-1\right)/n.$

(4) $$C^{\lambda }=g^{\lambda m}\cdot r^{n\lambda }\;\mathrm{mod}\;n^{2}=\left(1+\lambda mn\right)\;\mathrm{mod}\;n^{2}$$

Therefore,

(5) $$m=L\left(C^{\lambda }\;\mathrm{mod}\;n^{2}\right)/L\left(g^{\lambda }\;\mathrm{mod}\;n^{2}\right)\;\mathrm{mod}\;n.$$

1. Cryptographic Protocol of B
  Table 1 shows the cryptographic protocol of B. First, B binary-expands the quality data X and Y. Additionally, B encrypts the $x_{i}$ bit of X, the $y_{i}$ bit of Y, and $x_{i}⊕y_{i}$ using Formula (3). Then, it puts $Enc\left(x_{i}\right)$, $Enc(y_{i})$, $Enc(x_{i}⊕y_{i})$, and the public key into the blockchain, as shown in Figure 3 and Appendix A.
2. Calculation Protocol of S
  Table 2 shows the calculation protocol of S. S gets and saves $Enc(x_{i}⊕y_{i})$ and the public key from XOR CC, as seen in Figure 3. If there is a request from A, S uses the public key as a key to identify $Enc(x_{i}⊕y_{i})$ and sends the $Enc(x_{i}⊕y_{i})$ to A. S is a servicer that handles important XOR data in this B2B collaboration system.
3. Calculation Protocol of A
  Table 3 shows the calculation protocol of A. A gets $Enc\left(x_{i}\right)$, $Enc(y_{i}),$ and the public key from $Q_{\beta }$ CC, as seen in Figure 3. Additionally, A gets $Enc(x_{i}⊕y_{i})$ from S by sending a public key of t and t+1, where A wants to identify the change. A calculates those quality data using Formulas (1) and (2) while keeping them encrypted.
  In the proposed protocol for Step 3, it is necessary to calculate $Enc\left(-y_{i}\right)$ from $Enc\left(y_{i}\right)$. This proposed protocol uses Formula (6) from Paillier cryptography to calculate $Enc\left(-y_{i}\right)$.
  (6) $$C^{n-1}={\left(g^{m}.r^{n}\right)}^{n-1}\mathrm{mod}\;n^{2}=\left(1-mn\right).r^{n\left(n-1\right)}\mathrm{mod}\;n^{2}=g^{-m}.r^{n\left(n-1\right)}\mathrm{mod}\;n^{2}.$$
4. Decryption Protocol of B
  Table 4 shows the decryption protocol of B. Using the secret key and Formula (5), B decrypts $Enc(z_{i})$ and searches for the bit, where $z_{i}=0$.
5. Comparison Protocol of A
  Table 5 shows the comparison protocol of A. Finally, A receives i or knows that $z_{i}=0$ did not occur. If A receives i while using Formula (1), then X<Y can be determined. If A receives i while using Formula (2), then X>Y can be determined. Here, i is the first different bit when comparing X and Y from the most significant bit. Therefore, using this proposed protocol, A can grasp X<Y or X>Y and the scale of the difference between X and Y without knowing the numbers that X and Y represent; that is, A can confirm the change in quality data in the time series.

### 3.3. System Configuration

**Figure 4:**
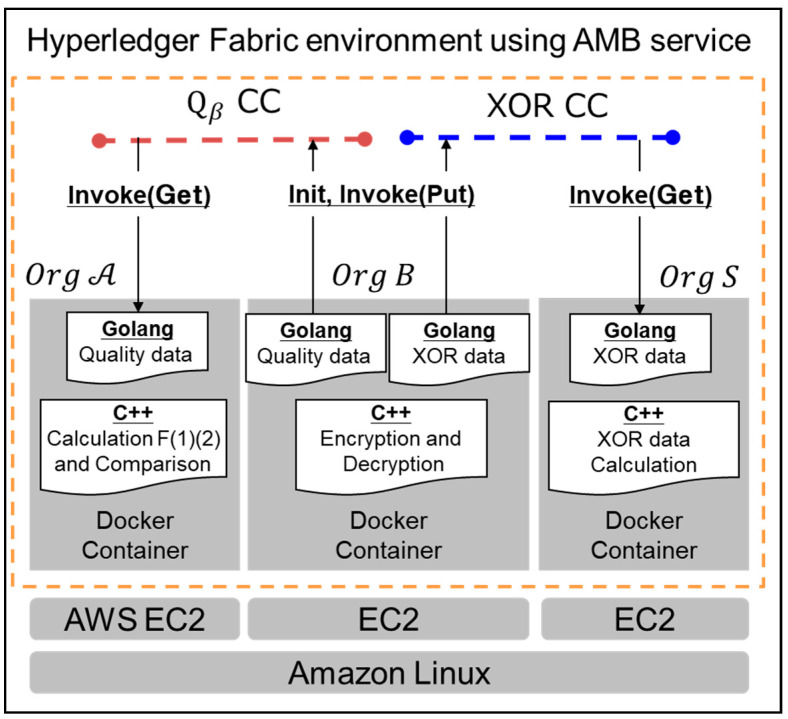
Configuration of proposed the B2B collaboration system.

The proposed system was constructed as shown in Figure 4. A company was set up as one organization in a docker container on the AWS EC2. A blockchain network was built using Hyperledger Fabric, which utilizes the Amazon Managed Blockchain (AMB) service. Hyperledger Fabric was adopted to build a private blockchain for companies in the SC. In order to efficiently calculate multi-length arithmetic, the encryption, decryption, and calculation of the data were programmed by C++. APIs for both putting data into and getting data from the blockchain were developed by Golang.

## 4. Evaluation Result of the Concrete Data-Collaboration’s Merits

**Figure 5:**
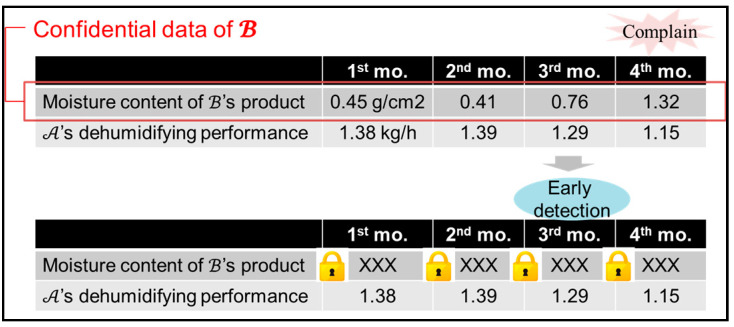
An actual business example of concrete data collaboration.

Finally, this paper considers the usefulness of the proposed system and protocol. In the actual case in Figure 5, manufacturer A provided dry dehumidifiers to the dry products of manufacturer B. One day, a complaint was made about the decreased performance of the dehumidifiers from B, and it turned out that the friction-reducing film, which should have been attached to the dehumidifying rotors, was not attached. It took four months from delivery for the performance reduction of be noticed. By applying the proposed protocol, B was able share product moisture data, which are confidential manufacturing data, with A in an encrypted manner. This paper evaluates whether A was able to notice the quality change at an early stage in the actual business setting.

**Figure 6:**
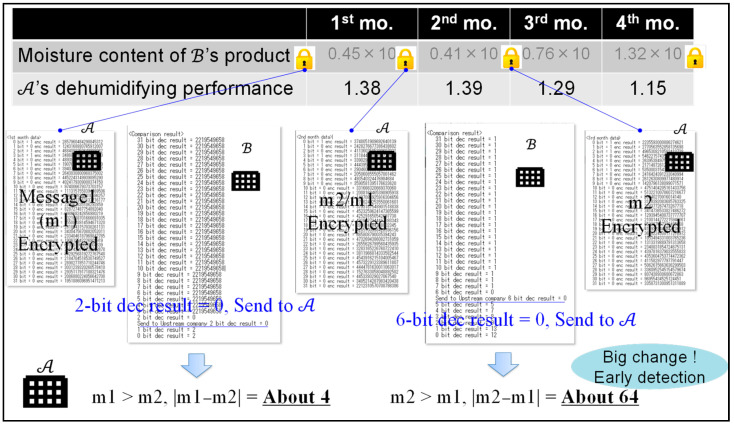
Evaluating the result of the concrete data collaboration.

Figure 6 shows the result of an experiment where the proposed protocol and system were applied. B was able to provide confidential moisture data encrypted as messages 1 and 2. Using the proposed system, A was able to detect the decreased performance in the third month by getting 10-times the encrypted moisture data from the quality chaincode and encrypted XOR data from the XOR chaincode so as to observe the changes in quality each month. In Paillier cryptography, message $m\in {\mathbb{Z}}_{n}$ is required, so the quality data are multiplied by 10. As can be seen in Figure 6, A does not know the specific numerical value of the quality data. This paper confirms the behavior and usefulness of the proposed system.

## 5. Safety Evaluation of the Proposed System

This paper does not consider the case where B maliciously puts incorrect quality data $Enc\left(x_{i}\right)$ and $Enc(y_{i})$ to $Q_{\beta }$ CC in Step 2 of Table 1. The incorrect quality data put by B means to disrupt the SC for their own material procurement. Such cases are nonsensical from a business perspective and are not worth considering.

In Paillier cryptography, since A does not know (p, q) and $g^{m}$ is masked by $r^{p\cdot q}$ in Formula (3), it is difficult to solve the discrete logarithmic problem in the exponential part of Formula (3). Thus, the message m cannot be identified [43].

In the proposed comparison protocol of the B2B collaboration system, since ciphertexts are encrypted using random number r in Formula (3), A cannot identify $x_{i}$ or $y_{i}$ by comparing $Enc\left(x_{i}\right)$, $Enc\left(y_{i}\right)$, and $Enc(x_{i}⊕y_{i})$, as can be seen in Figure 6.

In the B2B collaboration system, there is a risk that company C, participating in the quality channel, will impersonate A. Since C participates in the quality channel, C can get the public keys. If C intercepts A’s $Enc(z_{i})$, falsifies the encrypted data with Paillier cryptography, and sends it to B, A will not be able to grasp the change in quality. However, since C is also a company in the SC related to B’s products, when it is found that it is impersonating A, C will receive great punishment. That is, C will not be able to trade with any other company. Therefore, it is unlikely that spoofing by a company such as C will occur in the proposal system. Even if the encrypted data were to be leaked to a company that does not participate in a blockchain channel, it would not be tampered with unless the public key were leaked.

## 6. Conclusions

This paper proposed a secure comparison protocol for the sharing and grasping of changes to even highly sensitive data between companies by keeping the data encrypted. By implementing the protocol on a blockchain that connects companies in the industrial SC, this paper also proposed a B2B collaboration system. In a scenario with a business focusing on quality data, which is the most sensitive data in manufacturing, this paper showed that a company can grasp the change in the quality data of their business partner, while keeping the data encrypted, and subsequently feed that change back into its own manufacturing.

By using the proposed system, upstream companies will be able to grasp the quality changes of downstream companies. Combined with data from IoT sensors attached to their equipment, upstream companies will be able to proactively respond to the decreased performance of delivery equipment, which is directly linked to quality. In addition, small- and medium-sized manufacturers are said to lack sales resources, but the proposed system enables them to grasp the quality improvement needs of downstream companies in advance and reflect those needs in their own functional development. Upstream companies, many of which are large companies, do not disclose the quality value to other companies via encryption, and there is no concern that quality data will be tampered with by other companies by BC, so data can be provided with peace of mind. The social implementation of this research will create an ecosystem of collaboration among companies in the industrial SC, which will enable highly efficient manufacturing.

Note that the proposed protocol uses Paillier cryptography, which requires the use of a 6000-bit space for secure encryption and decryption. Investigating speed-up using elliptic cryptography or other methods is the goal for future research.

## Figures and Tables

**Table 1:** Encryption Protocol of B.

Step	Processing
1	B binary-expands X and Y and encrypts $x_{i}$ and $y_{i}$ (i = 0~k-1 bits) with the public key.
2	B puts $Enc\left(x_{i}\right)$, $Enc(y_{i})$, and the public key into the quality data chaincode $Q_{\beta }$ CC, as seen in Figure 3.
3	B calculates $Enc(x_{i}⊕y_{i})$ with the public key.
4	B puts $Enc(x_{i}⊕y_{i})$ and the public key into the XOR data chaincode XOR CC, as seen in Figure 3.

**Table 2:** Calculation Protocol of S.

Step	Processing
1	S gets and saves $Enc(x_{i}⊕y_{i})$ and the public key from the XOR data chaincode XOR CC, as seen in Figure 3.
2	S receives an XOR data request and a public key from A.
3	S identifies the $Enc(x_{i}⊕y_{i})$ that has the same public key sent by A in Step 2.
4	If there is an $Enc(x_{i}⊕y_{i})$, S sends the $Enc(x_{i}⊕y_{i})$ to A.

**Table 3:** Calculation Protocol of A.

Step	Processing
1	A gets $Enc\left(x_{i}\right)$, $Enc(y_{i})$, and the public key from the quality data chaincode $Q_{\beta }$ CC as seen in Figure 3.
2	A sends the public key to S and receives $Enc(x_{i}⊕y_{i})$ from S.
3	From the most significant bit, A calculates $Enc(z_{i})=Enc\left(x_{i}-y_{i}\pm 1+3\underset{j<i}{\sum }\left(x_{j}⊕y_{j}\right)\right)$.
4	A sends $Enc(z_{i})$ to B.

**Table 4:** Decryption Protocol of B.

Step	Processing
1	B receives $Enc(z_{i})$ from A.
2	B decrypts $Enc(z_{i})$ with the secret key.
3	If there is $z_{i}=0$, B sends i to A.

**Table 5:** Comparison Protocol of A.

Step	Processing
1	A receives i when $z_{i}=0$ or knows that $z_{i}=0$ did not occur.
2	If A receives i while using Formula (1), then X<Y can be determined. If A receives i while using Formula (2), then X>Y can be determined.
3	A checks the difference between the numbers at times t and t+1 by calculating $2^{i}.$

**Figure A1:**
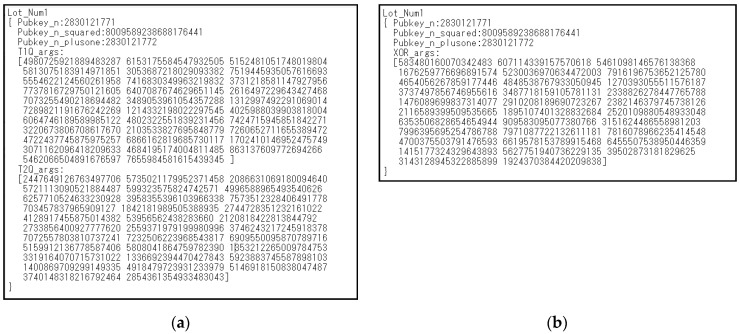
Data structure. (**a**) Data structure of $Q_{\beta }$ CC; (**b**) Data structure of XOR CC.

## Data Availability

Not applicable.

## Appendix A

Figure A1a,b shows the data structure of $Q_{\beta }$ CC and XOR CC, respectively. Quality data and XOR data are put into the BC with a common lot number and public key n to identify when they were put and the production lot data to which they correspond. In Figure A1, “Pubkey_n” is the Paillier cryptography public key. Figure A1 shows an example where n is 32 bits. “T1Q_args” and “T2Q_args” are the quality data of times t and t+1, respectively. “XOR_args” is the XOR data. The quality data and XOR data are bit-expanded and encrypted, respectively. The public key is updated every time, for a set of t and t+1.
